# Transcriptome Markers of Viral Persistence in Naturally-Infected Andes Virus (*Bunyaviridae*) Seropositive Long-Tailed Pygmy Rice Rats

**DOI:** 10.1371/journal.pone.0122935

**Published:** 2015-04-09

**Authors:** Corey L. Campbell, Fernando Torres-Perez, Mariana Acuna-Retamar, Tony Schountz

**Affiliations:** 1 Arthropod-borne and Infectious Diseases Laboratory, Department of Microbiology, Immunology and Pathology, Colorado State University, Fort Collins, Colorado, United States of America; 2 Instituto de Biología, Pontificia Universidad Católica de Valparaíso, Valparaíso, Chile; 3 Facultad de Cs Veterinarias y Pecuarias, Universidad de Chile, Santiago, Chile; University of Liverpool, UNITED KINGDOM

## Abstract

Long-tailed pygmy rice rats (*Oligoryzomys longicaudatus*) are principal reservoir hosts of Andes virus (ANDV) (*Bunyaviridae*), which causes most hantavirus cardiopulmonary syndrome cases in the Americas. To develop tools for the study of the ANDV-host interactions, we used RNA-Seq to generate a de novo transcriptome assembly. Splenic RNA from five rice rats captured in Chile, three of which were ANDV-infected, was used to generate an assembly of 66,173 annotated transcripts, including noncoding RNAs. Phylogenetic analysis of selected predicted proteins showed similarities to those of the North American deer mouse (*Peromyscus maniculatus*), the principal reservoir of Sin Nombre virus (SNV). One of the infected rice rats had about 50-fold more viral burden than the others, suggesting acute infection, whereas the remaining two had levels consistent with persistence. Differential expression analysis revealed distinct signatures among the infected rodents. The differences could be due to 1) variations in viral load, 2) dimorphic or reproductive differences in splenic homing of immune cells, or 3) factors of unknown etiology. In the two persistently infected rice rats, suppression of the JAK-STAT pathway at *Stat5b* and *Ccnot1*, elevation of *Casp1*, RIG-I pathway factors *Ppp1cc* and *Mff*, and increased FC receptor-like transcripts occurred. Caspase-1 and Stat5b activation pathways have been shown to stimulate T helper follicular cell (T_FH_) development in other species. These data are also consistent with reports suggestive of T_FH_ stimulation in deer mice experimentally infected with hantaviruses. In the remaining acutely infected rice rat, the apoptotic pathway marker *Cox6a1* was elevated, and putative anti-viral factors *Abcb1a*, *Fam46c*, *Spp1*, *Rxra*, *Rxrb*, *Trmp2* and *Trim58* were modulated. Transcripts for preproenkephalin (*Prenk*) were reduced, which may be predictive of an increased T cell activation threshold. Taken together, this transcriptome dataset will permit rigorous examination of rice rat-ANDV interactions and may lead to better understanding of virus ecology.

## Introduction

Several species of hantaviruses (family *Bunyaviridae*) have been identified as human pathogens (reviewed in [1,2]), and some cause hantavirus cardiopulmonary syndrome (HCPS), a disease that has killed hundreds in South America [3,4]. The long-tailed pygmy rice rat (*Oligoryzomys longicaudatus*, “rice rat”) is found throughout most of southern South America and is the principal reservoir of Andes virus (ANDV) [5]. The species belongs to the Rodentia subfamily Sigmodontinae, as does the North American reservoir of Sin Nombre virus, the deer mouse (*Peromyscus maniculatus*) [6,7]. Pathogenic hantaviruses circulate in rodent reservoirs without causing substantial disease, and the reservoirs are thought to remain infected with hantavirus for life (reviewed in [2]), despite the production of neutralizing antibodies [6]. Rice rats also may be a reservoir of the Lyme borreliosis group member *Borrelia chilensis* [8]. Despite the importance of the rice rat as a reservoir host for important human pathogens, little is known about its host response during infections.

Experimental hantavirus infections of natural reservoir hosts have been largely limited to one New World species, deer mice infected with SNV [6,9–11], and two Old World hantavirus reservoirs [12–14]. No substantive experimental work on ANDV infection of rice rats has been conducted, thus it is challenging to assess the host response to identify differences and similarities with other hantavirus reservoirs. Moreover, the only small animal pathology model of hantavirus disease uses Syrian hamsters (*Mesocricetus auratus*), in which ANDV or Maporal virus causes signs resembling HCPS [15]. Unlike reservoir host infections, ANDV causes fatal disease in hamsters, with signs of vascular and respiratory pathology followed by death within two weeks [16]. A robust innate immune response, with expression of *Stat2* and other anti-viral factors, and a modest antibody response, occurs late in infection (9 to 11 days-post-infection (dpi)) before death occurs. In contrast, experimental infection of Syrian hamsters with wild-type SNV results in a robust adaptive immune response that occurs earlier in infection, followed by virus clearance [16,17].

To increase understanding of long-tailed pygmy rice rat genetics, ANDV–reservoir interactions and the markers of infection, we performed RNA-seq analysis of spleens from five rice rats collected in Chile [18]. The major goals of this work were to provide a sequence dataset for this species and to identify differences in transcriptional profiles associated with ANDV infection. Three of the five rice rats were seropositive with detectable viral RNA. Reference-independent sequence assembly and estimation of transcript abundance allowed quantitative assessment of RNA-seq data [19,20]. Subsequently, fastq reads of ANDV- infected (n = 3) and uninfected rice rat spleens (n = 2) were subjected to differential expression analysis to identify host transcripts that could be pertinent to the establishment of a persistent infection. Finally, phylogenetic analysis was performed to define the relationship of rice rats to other mammals.

## Materials and Methods

### Ethics Statement

All methods for trapping and processing rice rats were approved by the Institutional Bioethics Committee, Pontificia Universidad Católica de Valparaíso, Chile. The permit for trapping rodents was granted by the Servicio Agrícola y Ganadero (permit #6134, 9 Sep 2011), Chile. This study did not involve endangered or protected species.

### Rodent Collection

Rice rats were live-trapped using Sherman traps near Villarica, Region IX, Chile (coordinates- 39°25’S, 71°45’W), November 18–22, 2011 [18,21]. Rodents were anesthetized with isoflurane and bled from the retroorbital plexus for subsequent antibody testing. Anesthetized rodents were euthanized by cervical dislocation, followed by necropsy. Spleens were flash-frozen in liquid nitrogen in the field for transport, then stored at -80°C at Pontificia Universidad Católica de Valparaíso prior to dry ice shipment to Colorado State University. Rice rats #18 (RR18) (pregnant), #29 (RR29) (scrotal male) and #31 (RR31) (adult male) were seropositive. Rice rats #20 (RR20) (adult male) and #30 (RR30) (lactating) were seronegative.

### Serology and Determination of Viral RNA Load

ELISA detection of anti-ANDV N antibodies (Ab) was reported previously [18,22]. Viral RNA was quantitated using previously published primers and a modification of a real-time PCR assay for detection of the ANDV S segment [11]. Briefly, dilutions of ANDV (10^6^, 10^4^, 10^2^ TCID_50_) were prepared for RNA extractions and used as standards. RNA was extracted from spleens (described below), amplified using a One-Step SYBR Green RT-PCR kit (Qiagen) on a Bio-Rad MyiQ thermal cycler and copy number estimated using linear regression.

### RNA isolation and Sequencing

Total RNA was isolated from spleens using RNeasy kit (Qiagen). Spleens were homogenized in RLT buffer containing beta mercaptoethanol and stainless steel beads then passed over QiaShredder columns per manufacturer’s instructions. RNA-seq libraries were prepared from 500 ng total RNA using Ribo-Zero (Illumina) library preparation methods and the manufacturer’s recommended procedure. Five spleen RNA-Seq libraries were prepared separately and pooled on a single HiSeq 2000 (Illumina) lane for paired end 2x100nt sequencing. Library preparation and sequencing were performed at the University of Colorado Medical Center core facility. All raw fastq sequences are available at the NCBI sequence read archive under BioProject ID PRJNA258076.

### Bioinformatics

Fastq files were quality and adapter-trimmed using default parameters of Trimmomatic version 0.30 [23]. Using a reference-independent protocol, reads were assembled into contigs using the Trinity package (version 2013-02-25) and the following parameters [19]; JM 350G, CPU 24, SS_lib_type RF, kmer = 3. Kmer = 3 was an option that required a kmer coverage of 3 prior to transcript extension; this option was used as a preemptive measure for transcript error correction in a manner similar to the kmer spectrum-based approach recommended in Yang *et al*. [24]. A total of 158,078 contigs were produced from the assembly. All sequences have been archived at the NCBI Sequence Read archive (SRA, http://www.ncbi.nlm.nih.gov/sra) under accession number PRJNA258086. The transcriptome is also available for the design of PCR primers, using the Primer BLAST Tool at the following website (http://dna.publichealth.uga.edu/BlastPrimer/BlastPrimer.php) [25].

The contigs were representative of unique transcripts, independent isoforms and paralogs. The contig N50 score, which is a statistic representing a weighted average transcript length, was 2603 (Table 1). For all rice rat transcripts identified, a conservative approach was taken to orthology assignments. The house mouse (*Mus musculus*) mRNA RefSeq list was used as the primary reference, due to its detailed functional annotation. In addition, wild *Mus musculus* with hantavirus-specific antibodies have been identified (reviewed in ([7]). Therefore we expected that orthology assignments based *Mus musculus* RefSeq would be the most appropriate for this study. The assembled reads were annotated by BLASTx of the *Mus musculus* mRNA RefSeq database, using an Evalue limit of 10^–20^ [26]. *Mus musculus* RefSeq mRNA (mm9) was obtained from ftp://hgdownload.cse.ucsc.edu/goldenPath/mm9/bigZips/. Other organisms (BLAST nr database) were used as references for BLAST searches for differentially expressed genes when no significant ortholog was identified in the *Mus musculus* RefSeq reference. Further annotation is described below.

**Table 1:** Library Details.

	Totals	Transcripts
**Raw reads**	364.5 million	
**Trimmed QC reads**	319.1 million	
**Total assembled reads**	158,078	
**N50 of assembly**	2603	
**# RefSeq BLASTx hits**		66,315
**Unique RefSeq hits**		14,364

Gene annotation information for *Mus musculus* was downloaded from Jackson Laboratories (http://www.informatics.jax.org/). Gene orthologs and functional categories were determined from annotation of RefSeq genes, as well as the result of NCBI DAVID pathways KEGG pathway annotation (http://david.abcc.ncifcrf.gov/). All sequence alignments and phylogenetic analyses were performed in the Geneious package version 7.0.4, using the PHYML plug-in [27]. Each alignment was subjected to 1000 bootstrap replicates to obtain the final trees. The Jones-Taylor-Thornton model of amino acid substitution was applied [28]. Immune response pathway transcript annotation was performed in Reactome (version 4.1.1) [29,30].

### Immunoglobulin and T Cell Receptor Identification

The *de novo* Trinity assembly was also used to search the IMGT databank to identify immunoglobulin heavy and light chain polypeptides, as well as T cell receptor chain polypeptides (ngKLAST v.r. 4.5, Korilog SARL, France, http://www.korilog.com). The parameters were as follows, E-value threshold: 10, BLOSUM 62 matrix and default gap penalty. Hit annotation data were imported into a database (Filemaker Pro 12) to identify Ig and TCR transcripts. Transcripts with putative complete 5’ ends (i.e., ATG start codons) were analyzed for V(D)J segments with the NCBI IGBLAST tool (http://www.ncbi.nlm.nih.gov/igblast/) to identify gene segment orthologs from the laboratory mouse [31].

### Differential Expression Analysis

Individual rice rat transcript assemblies were screened for other infectious agent sequences to exclude the possibility that differential expression was influenced by co-infection with other agents. The *de novo* Trinity assembly was used to search the NCBI non-redundant database for all bacterial and viral sequences, using an E value cut-off of Evalue limit of 10^–40^, BLOSUM 62 matrix, and default gap penalty (ngKLAST). No evidence of bacterial or parasite infection was found among the seropositive spleens that was not also found in the seronegative samples. In addition, all retrovirus and DNA virus sequences found in RR29 were also present in seronegative controls and likely represented endogenous retroviruses or paralogs of DNA viruses.

Within the Trinity package, fastq reads from each of 5 spleen samples were subjected to RSEM estimation of transcript abundance by aligning them against the Trinity-assembled contigs. Estimated count data from RSEM was used as input for DESeq differential expression analysis [32]. For a given transcript, a minimum of 200 reads, when summed across all biological replicates, were required for inclusion in DESeq analysis. In addition, a multiple testing adjustment (FDR) *p* value <0.05 cut-off was also applied [33]. For four rice rats (RR18, RR20, RR30, RR31), DESeq (version 1.1.6) default size factors normalization was applied, as well as the following dispersion estimation parameters: method = "pooled", sharingMode = "maximum", fitType = "local". Due to a higher viral load, RR29 was compared to seronegative controls in a separate comparison (parameters: method = "blind", sharingMode = "fit-only", fitType = "local"). DESeq is able to evaluate the single rice rat data input in comparison to the two uninfected controls, because variance estimates were adjusted by treating all samples as replicates. The fit-only parameter removes the DESeq algorithms’ ability to remove outliers; therefore, manual filtering was required. Heat maps were generated using pheatmap and (clustering_distance_cols = "euclidean") within the R Bioconductor statistical package (http://www.r-project.org).

## Results and Discussion

### Infection status and Transcriptome assembly

**Fig 1:**
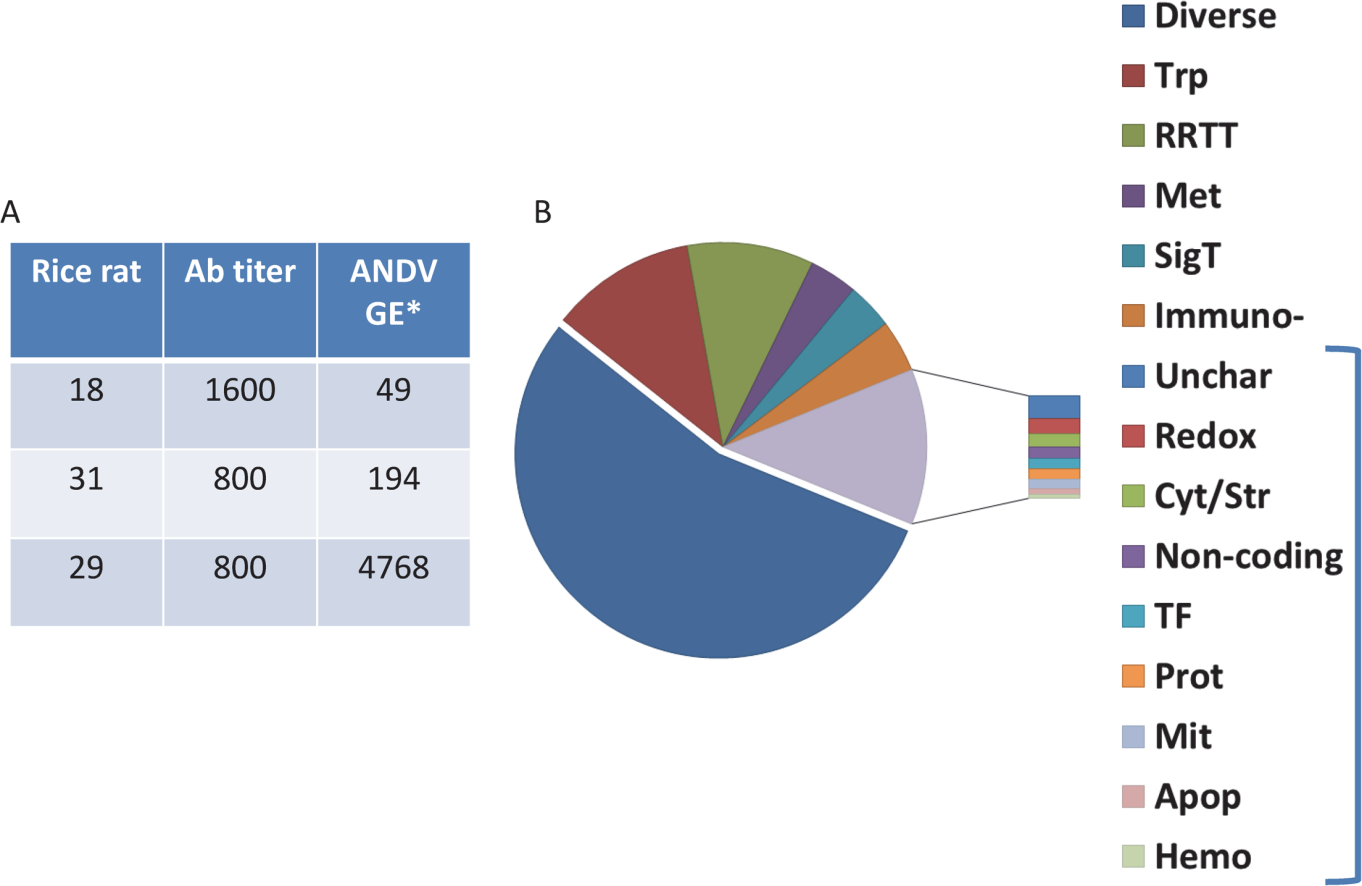
Infection status of seropositive rice rats and spleen transcriptome functional groups. A) Seropositive rice rat ANDV qRT-PCR results. *, GE, genome equivalents ANDV S segment. Seronegative rice rats were confirmed to be ANDV virus negative by qRT-PCR. ELISA antibody titers are reproduced from a previous report [18]. B) All unique annotated transcript orthologs (n = 16,708) are represented by functional category. Diverse; Trp, intra-, inter-cellular and cellular transport functions; ‘RRTT’, (DNA) repair, replication, transcription, translation; ‘SigT’, signal transduction; ‘Met’, metabolism; ‘Unchar’ unknown or uncharacterized; ‘Immuno-’, immunomodulatory; ‘ReDox’, reduction/oxidation; 'Cyt/Str', cytoskeletal/structural; ‘Non-coding’ non-coding regulatory RNAs or pseudogenes; ‘TF’, transcription factors or suppressors; ‘Prot’, proteolysis or 26S proteasome function; ‘Mit’, mitochondrial function; ‘Met’, metabolism; ‘Apop’, apoptosis; ‘Hemo’ hemostasis (factors controlling angiogenesis and hematopoiesis). Bracket indicates exploded functional groups, each representing less than 3% of the total.

As previously reported, RR18, RR29, RR31 were seropositive for hantavirus nucleocapsid as determined by ELISA [18]. Spleens from five rice rats were processed for individual RNA-seq deep sequencing libraries. RR29 was the only rice rat among the five sequenced that showed the presence of ANDV (M segment) in the RNA-Seq data (S1 Table). However, the infection status for all three seropositive animals was confirmed by qRT-PCR of S segment genome equivalents (GE) (Fig 1A), indicating greater sensitivity of PCR relative to RNA-seq. The resulting 364 million pooled paired end reads were used for reference independent *de novo* sequence assembly using the Trinity package [19]. Among all rice rat spleen libraries, 158,078 contigs were assembled (Table 1). Of these, 66,173 independent transcript isoforms and non-coding RNAs were identified (BLASTx ≤E^-20^); about one half of all contigs showed very low confidence similarity (BLASTx, >E^-20^) to RefSeq or Genbank orthologs (S1 Table). Of the annotated transcripts, 16,708 unique genes were identified and graphed according to functional group (Fig 1B). Of these, there were a number of long non-coding RNAs, 14 miRNA genes and 34 small nucleolar non-coding RNAs. In addition to the more abundant functional categories, such as intra- and inter-cellular transport (Trp), DNA repair/replication/transcription/translation (RRTT), and the immuno-modulatory (Immuno-) category, rare transcripts associated with hemopoiesis and angiogenesis were also identified (Hemo, hemostasis).

### Immune system transcripts

**Fig 2:**
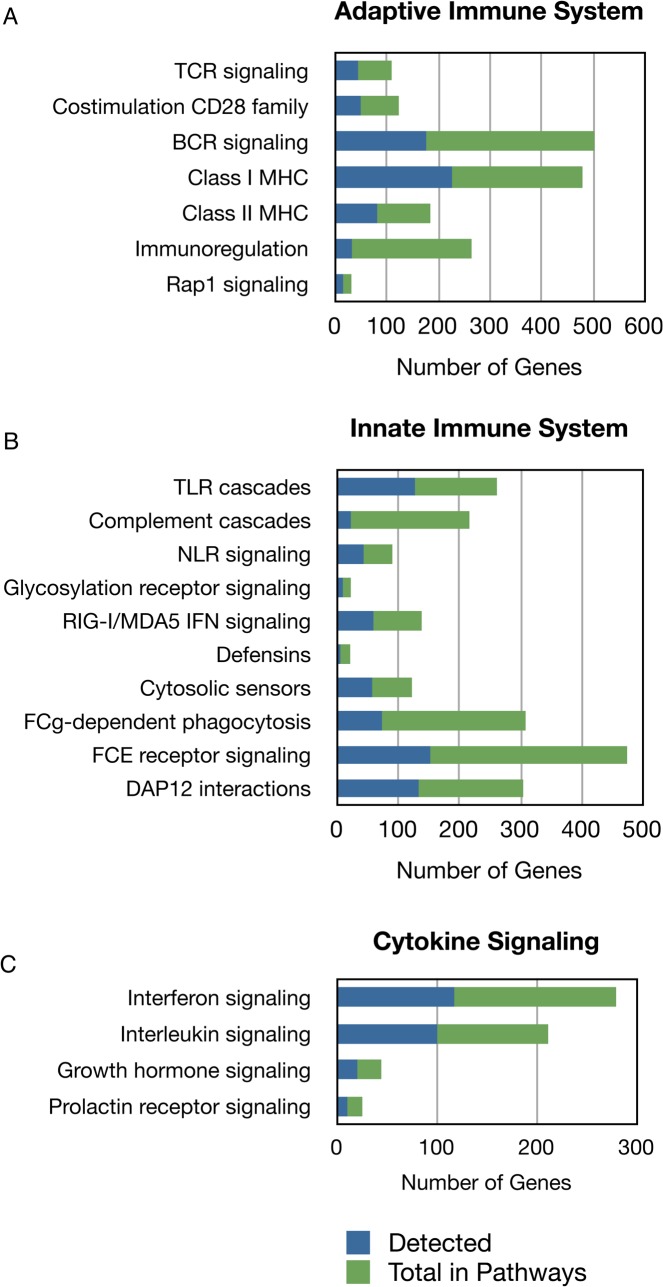
Immune response effectors identified using the Reactome pathways analysis tool [29,30]. Unique transcripts present in the RNA-Seq assembly were assigned to A) Adaptive immune, B) Innate immune, and C) Cytokine signaling pathways. Blue portion of bar indicates the rice rat genes per pathway category in the Reactome database; green bar indicates the total number of genes per Reactome category not represented in our dataset.

About 4.0% of the unique transcripts from the assembly represent orthologs of immunomodulatory functional groups (Fig 1B). All major categories of adaptive and innate immune responses were identified (Fig 2; S2 Table). Of the 16,708 unique genes, 895 represented immune system-related orthologs found in the curated Reactome database, which is equivalent to 71% of all immune genes in the database [29]. Of these 895 rice rat orthologs, 507 of 770 in this category were predicted to be associated with adaptive immune responses, 427 of 688 in this category were associated with innate immune responses, and 216 of 276 in this category were in cytokine signaling pathways. Several genes, for example, those involved in cytokine signaling events, were predicted to be involved in both adaptive and innate immunity categories, and thus were counted in all categories. The predicted functional subcategories for these immune response genes are shown in S2 Table. Transcripts encoding immunoglobulins and T cell receptors were also present in the assembly, including those for IgM, IgD, IgG1, IgG2a, Igk, TCRα and TCRβ. Notably, IgG2b, IgG3, IgA, IgE, Igλ, TCRγ or TCRδ sequences were not identified. Some transcripts had complete V(D)J sequences and analysis of these revealed highly similar sequences to house mouse Ig and TCR gene segments (S1 Table) [31].

To estimate evolutionary distances between the rice rat and other hantavirus hosts and reservoirs, we examined selected predicted proteins of the rice rat immune response in comparison to deer mouse, Syrian hamster, house mouse, Norway rat (*Rattus norvegicus*), the reservoir of Seoul hantavirus, and human orthologs [12,16]. Maximum likelihood phylogenetic analysis of 1) signal transducer and activator of transcription peptides 2 and 5 (Stat2, Stat5b), 2) interleukin-1beta (Il1b) and complement factor B, and 3) nitric oxide synthase 2 (Nos2) and alpha-zinc-2-glycoprotein 1 (Azgp1), indicated that, with the exception of Nos2 and Stat5b, rice rat orthologs clustered with either hamster or deer mouse rather than the house mouse (S1 Fig). In addition, a lack of bootstrap support for Stat5b suggested that it is variable among all species tested.

### Non-coding RNAs

About 1.3% of the unique assembled annotated transcripts represent non-coding RNAs or pseudogenes (Fig 1B, Table 2). Among these, 14 unique microRNA genes were identified. Most have not been associated with virus infection; however three, miR-122, miR-324 and let-7, have been identified in studies of host responses to viruses [34–36]. mIR-122 may affect type I IFN expression in tissues of immune origin by blocking suppression by cytokine signaling [37]. Importantly, mir-122 participates in natural killer (NK) cell activation by increasing expression of CD69, an activation receptor for NK cells, as well as increasing secretion of IFNγ [38]. Although mIR-122 has been reported to be a liver-specific miRNA; its presence in spleen could be due to species differences or the migration of leukocytes between organs. Let-7 family miRNAs have also been shown to have immunomodulatory function. All let-7 family members show anti-viral properties during flavivirus infection [34], and in human cell culture, let7-c targets IL-10 to reduce IL-10 expression levels [39]. In addition, mIR-324 levels are enriched in the presence of IFNα, although the implications of this for the immune response are not well understood [36].

**Table 2:** Non-coding RNAs.

Type	# Unique Transcripts		Reference, anti-viral function
**miRNA gene**	**14**		
microRNA 1199			
microRNA 122a			Pedersen et al., Nature, 2007 and others
microRNA 1898			
microRNA 1983			
microRNA 25			
microRNA 3068			
microRNA 3074–1			
microRNA 324			Zhang et al., PLoS One, 2013
microRNA 341			
microRNA 425			
microRNA 450b			
microRNA 486			
microRNA 7–1			
microRNA let7d			Cheng, J. Virology, 2013
**rRNA**	**1**		
**small nucleolar RNA**	**59**		
**small nuclear RNA**	**2**		
**other non-coding**	**324**		

A number of long non-coding RNAs (lncRNAs) were also found that align with high similarity to those in the house mouse RefSeq RNA geneset (Table 2). LncRNAs modulate the expression of target genes using a variety of mechanisms [40,41]. LncRNA categories are also varied and range from long intergenic types (lincRNA) to transcribed pseudogenes (Table 2). Anti-sense lncRNAs may regulate target genes in *cis*, following amphipathic transcription from the same promoter as the target mRNA [41]. A second type that also regulates genes in *cis* is the enhancer lncRNA (eRNA) [42]. Although the modes of action of lncRNAs are not well understood, they appear to occur primarily through RNA-protein interactions [43]. Some of these may specifically target spliceosome components to regulate alternate splicing machinery [44].

### Differential Expression Analysis

In a previous report of experimental infection of deer mice with SNV, viral loads peaked between 10 to 15 days post-infection, and persisted at lower levels thereafter [9]. Similarly, as reservoirs of ANDV, rice rats are expected to be infected for life; therefore, the presumptive designation of ‘persistently infected’ was given to RR18 and RR31. This rationale was based on the observation of low levels of viral RNA and the presence of ANDV-specific antibodies. In contrast, RR29 may have had an acute infection due to the substantially higher viral load compared to the other two rice rats and presence of ANDV in the RNA-Seq data (Fig 1A, S1 Table). RNA-seq data from the seropositive, persistently infected rice rats (RR18, RR31) was analyzed by RSEM and DESeq differential expression analysis (R, Bioconductor) to calculate the log_2_ fold-change (Log_2_FC) in transcript abundance, compared to seronegative controls (n = 2). Similarly, RR29 was also compared against seronegative controls in a separate experiment. Notably, the differential expression analysis in this report was performed on data from a small number of spleens from naturally-infected outbred rice rats of unknown incidence. Therefore, until additional corroborating experiments are performed, the possibility must be considered that additional mitigating factors may have played a role in the distinct expression pattern found for RR29.

One possibility is that reproductive status differences among the individuals could have contributed to differences in the results. Although both sexes were represented among the seropositive group and seronegative controls, individual variability in these differences, as well as unknown factors, could have contributed to the differences found. For example, seronegative RR30 was lactating, and seropositive RR18 was pregnant. In contrast, seropositive RR29 was the only scrotal male. Known dimorphic differences exist in the immune cell repertoire and cytokine response of rodent spleens [45–47]. It’s possible that the majority of the distinct features of RR29 and RR18/RR31 responses, described below, were due to effects of gonadal steroids or other hormonal differences rather than ANDV per se.

**Fig 3:**
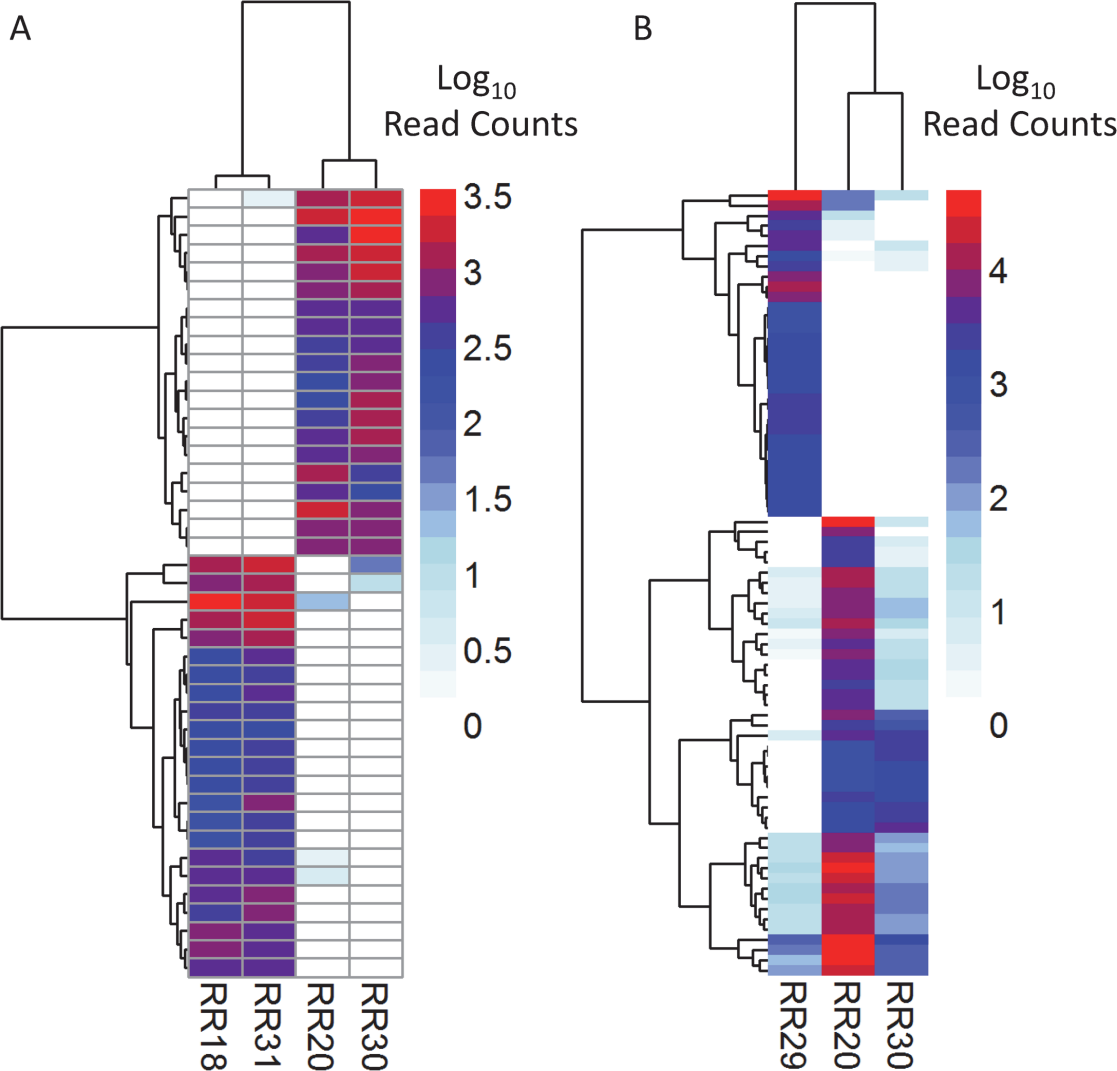
Heatmap of differentially expressed transcripts. A) Persistently infected rice rat (RR18, RR31) Log_10_ read counts compared to seronegative controls (RR20, RR30). B) RR29 Log_10_ read counts, relative to those of seronegative controls. Scale shows Log_10_ read count values. Heatmaps made with pheatmap (R, Bioconductor), using euclidean clustering distances.

In the comparison of RR18 and RR31 against seronegative controls, 43 differentially expressed transcripts were identified (FDR, *p*<0.05) (Fig 3A, S3 Table). Of these, 17 transcripts were completely absent in seronegative animals but present in seropositive. One such transcript, *Casp1*, is a cytokine maturation caspase that activates pro-inflammatory components [48]. Its expression may be stimulated as part of the anti-viral sensor response system induced by Toll-like receptors [49]. As part of the inflammasome complex, caspase-1 stimulates activation of interleukin-1β, which participates in a variety of effects, such as cellular proliferation and differentiation, as well as activation of IL-18 (reviewed in [50]). Moreover, caspase-1 can also trigger a Th2-biased immune response [51].

**Fig 4:**
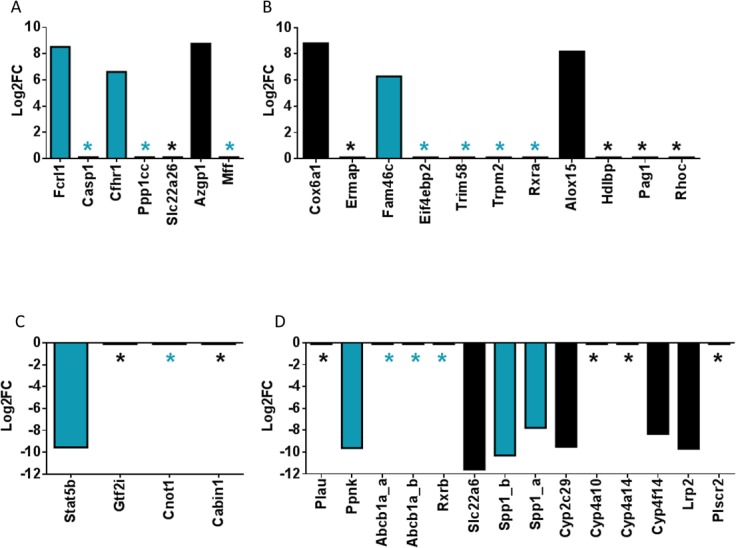
Differential expression signatures of immune response-related and lipid metabolism transcripts. Transcripts were subjected to RSEM abundance estimation and DESeq estimation of fold-change, based on a negative binomial distribution (FDR, *p* <0.05). Stars indicate presence in the seropositive group and absence in seronegative controls (top panel) or complete abrogation of the indicated transcript in seropositive animal spleens (bottom panel). Immune response transcripts are marked in blue. The remaining transcripts code for those predicted to be involved in lipid metabolism (*Cyp* gene family, *Lrp2*, *Pag1*, *Plscr2*, *Azgp1*, *Alox15* and *Hdlbp*), hemostasis (Ermap, Plau, *Gtf2i*, *Rhoc*), apoptotic proteins (*Cox6A1*), or are closely related to proteins known to affect immune function (*Slc22a6*, *Cabin1*). A and C) Selected differentially expressed transcripts in RR18/RR31 relative to seronegative controls. B and D) Selected differentially expressed transcripts in RR29 relative to seronegative controls.

Two modulated transcripts that were exclusively present in persistently infected rice rats were predicted to be associated with the RIG-I signaling pathway. The catalytic subunit of phosphatase 1, gamma isoform, (*Ppp1cc*) was elevated (Fig 4A). Importantly, Ppp1cc dephosphorylates RNA sensors, such as RIG-I and MDA5 to induce interferon-β (IFNβ) production [52]. Ppp1cc has also been shown in mouse vascular smooth muscle cells to have a protective effect by preventing p53 and Jun kinase-induced apoptosis [53]. Secondly, mitochondrial fission factor (*Mff*), was also differentially expressed in the same manner as *Ppp1cc* (Fig 4A). *Mff* is elevated during flavivirus infection of human cell culture, which could be evidence of an anti-viral mitophagic response [54]. In addition, an FC receptor-like transcript (*Fcrl1*) was also enriched over 8-fold Log_2_FC compared to uninfected rice rats (Fig 4A); *Fcrl1* enrichment is indicative of B cell activation [55]. This observation is supportive of a shift toward an adaptive immune response. Other evidence for immune response activation was observed in the over 6-fold Log_2_FC enrichment of Complement factor H-related (*Cfhr1*) (Fig 4A). *Cfhr1* is a regulator of the complement cascade and directly binds apoptotic cells (reviewed in [56]). A transcript encoding zinc alpha-2-glycoprotein 1 (*Azgp1*) showed a log_2_FC enrichment of 8.8 in infected rice rats. Azgp1 is an adipokine and physically interacts with hepatitis C viral protein F, which is a stimulator of T cells [57,58].

Twenty transcripts were depleted in the persistent infection group; of these, *Stat5b* is the best characterized (S3 Table, Fig 4C). Depletion of Stat5b is associated with maturation of T helper follicular cells (T_FH_) [59]. Moreover, signatures of a T_FH_ cell response were also reported for deer mice experimentally-infected with ANDV and, to a lesser extent, SNV [11]. The remaining nineteen depleted transcripts were absent in persistently infected rice rats. One abrogated transcript encodes nischarin (*Nisch*), which is associated with apoptosis of neuronal cells [60] (S3 Table). A second abrogated mRNA, *Cnot1*, encodes subunit 1 of the CCR4-NOT transcription complex, which is proposed to stimulate JAK/STAT pathway-dependent gene expression in humans and increase IFNγ production (S3 Table, Fig 4C) [61]. Its elimination could indicate suppression of at least one prong of the JAK/STAT response and a limitation in IFNγ levels. Lastly, calcineurin-binding protein 1 (*Cabin1*) is also abrogated in infected rice rats (S3 Table, Fig 4C); its presence is required for NFAT-mediated induction of T cell apoptosis [62]. It is an inhibitor of the calcineurin-NFAT (nuclear factor of activated T cells) pathway [63,64].

An important angiogenic marker, *Gtf2i*, is also abrogated in the persistent infection group (S3 Table, Fig 4C). Gtf2i has an important role in angiogenesis of microvascular cells by regulating the expression of VEGFR, the receptor for vascular endothelial growth factor (VEGF) [65]. In human microvascular cell culture, siRNA knockdown of *Gtf2i* resulted in stimulated expression of VEGFR, which is expected to stimulate capillary network formation. Therefore, our data suggest that angiogenic mechanisms may also be affected in rice rats persistently infected with ANDV, although no gross evidence of pathology was observed.

Differential expression of RR29 showed a strikingly different pattern from that of the putative persistently infected group. More transcripts (n = 77), in an expanded array of functional categories were differentially expressed (Figs 3B and 4, S3 Table); however any conclusions drawn from RR29 must be considered provisional until functional validation can be performed. Indeed, evidence of gonadal steroid expression differences were identified in RR29 compared to seronegative, non-scrotal negative controls (S3 Table).

**Fig 5:**
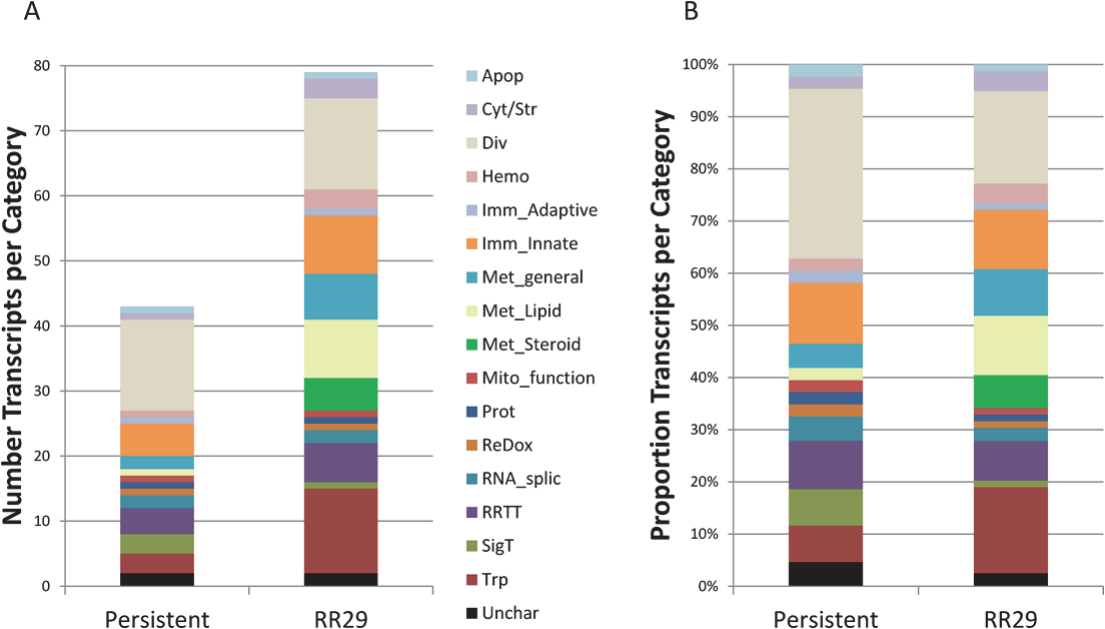
Transcript functional category differences in seropositive rice rats versus RR29. Transcript functional categories for each condition were plotted according to the number of transcripts in each category (A) or proportion of reads per category (B). ‘Apop’, apoptosis; 'Cyt/Str', cytoskeletal/structural; ‘Div’, Diverse; ‘Hemo’ hemostasis (includes factors controlling angiogenesis and hematopoiesis); ‘Imm_Adaptive’, adaptive immune response; ‘Imm_Innate’, innate immune response; ‘Met_general’, general metabolism; ‘Met_Lipid’, lipid metabolism; ‘Met_Steroid’, steroid metabolism; ‘Mito_function’, mitochondrial function; ‘Prot’, proteolysis or 26S proteasome function; ‘Redox’, reduction/oxidation; ‘RNA_splic’, mRNA splicing; ‘RRTT’, (DNA) repair, replication, transcription, translation; ‘SigT’, signal transduction; ‘Trp’, transport; ‘Unchar’ unknown or uncharacterized. The immune response and hemostasis functional groups were given priority for classification of multi-functional components.

Ratios of immune transcript functional categories differed for each group (Fig 5). For example, there was a higher proportion of transport and metabolic function transcripts affected in RR29 than in the persistent infection group (Fig 5B). The metabolic functional group was divided into lipid, steroid and general subgroups to more precisely delineate the differences; this revealed that more transcripts encoding lipid biosynthesis factors were modulated in RR29 compared to the persistently infected group. In addition, a different variety of immune response transcripts was modulated in RR29 compared to the persistently infected group, although the overall proportion of immune response transcripts was unaltered (Fig 5B). For example, retinoid X receptor alpha (*Rxra*), *Trpm2*, and *Trim58* were present in RR29 but absent in uninfected controls (Fig 4B). Enriched *RXRA* expression is predicted to promote chemokine stimulation and increased engulfment of apoptotic cells [66,67]. *Trpm2* and *Trim58* are not as well characterized but are predicted to be involved in the immune response. Interestingly, *Rxra* and *Rxrb* (retinoid X receptor beta) showed contrasting levels of enrichment and depletion, respectively. Rxrb is proposed to be involved in regulation of MHC class I and II expression [68]. An apoptotic marker, *Cox6a* was also significantly enriched in RR29, whereas in the persistently infected group, apoptotic process-associated transcripts (*Ppp1cc*, *Nisch*, *Cabin1*) were modulated in a manner predicted to be protective. In addition, *Fam46C*, an IFN-stimulated gene, was also enriched; interestingly, Fam46C has been shown to stimulate flavivirus replication in *STAT1* ^-/-^ human cell culture [69].

Several immune response transcripts were significantly depleted in RR29 (Fig 4D). For example, two isoforms of *Spp1* (secreted phosphoprotein 1) were depleted. The predicted effect of Spp1 is dependent on the intracellular location of the translated product; however, in general, this protein is predicted to promote signal transduction of immune response factors [70]. The transcript encoding preproenkephalin (*Ppnk*), a neuroendocrine hormone, was also significantly depleted. *Ppnk* depletion is predictive of a significant increase in the threshold for T cell activation [71]. Moreover, two isoforms of a transcript coding for an ATP-binding cassette subfamily B (*Abcb1a*) were also depleted; the lack of Abcb1a protein has been associated with increased susceptibility to virus infections [72]. The combined depletion of *Abcb1a*, *Spp1* and *Ppnk* are consistent with the hypothesis that T cell signaling and activity were suppressed in RR29.

Several markers of lipid metabolism were modulated in RR29, as well. The *Alox15* transcript, one of two enriched lipid metabolism transcripts, codes for arachidonate 15-lipoxygenase. Lipidomic studies of influenza infection models suggest that enriched *Alox15* levels occur during infection with a low pathogenicity virus [73]. In addition, *Hdlbp* (high density lipoprotein [HDL] binding protein) and *Pag1* (phosphoprotein associated with glycosphingolipid microdomains 1) were also present in RR29 but absent in seronegative controls. Enrichment of *Alox15*, *Pag1* and *Hdlbp* was coupled with significant depletion of a number of lipid metabolism-associated *Cyp* transcripts, as well as *Lrp2* (low density lipoprotein receptor-related protein 2) and *Plscr2* (phospholipid scramblase 2) (Fig 4D).

Markers of hemostasis were also affected in RR29, as they were in the persistently infected group. *Rhoc*, ras homolog gene family member C, was enriched and is predicted to modulate angiogenesis and increase VEGF levels *in vitro* (Fig 4B) [74]. Further evidence of effects on the vascular system was seen in the enrichment of *Ermap*, erythroblast membrane-associated protein. In contrast, plasminogen activator urokinase (*Plau*) levels were depleted. Interestingly, secretion of the Plau receptor (*Plaur*) in humans has been proposed as diagnostic marker for other hemorrhagic diseases, such as Crimean-Congo hemorrhagic fever [75].

Our hypothesis that RR29 had an acute ANDV infection was supported by multiple lines of evidence. For example, RR29 had 3.7 logs ANDV GE present per mg tissue (Fig 1A), which was within the detection limits of RNA-Seq (S1 Table). This rice rat also showed depletion of the T cell signaling/response components, *Abcb1a*, *Spp1* and *Ppnk* and no signatures of adaptive immunity, as were observed for RR18/RR31. Moreover, modulation of lipid metabolism and angiogenic transcripts was also observed.

The classical pathophysiological response in susceptible hosts exhibiting signs of a viral hemorrhagic fever, such as HCPS, is an increase in microvascular permeability [76,77]. In the case of hantaviruses and flaviviruses, this permeability is thought to be caused by a cytokine storm rather than virus-induced injury (reviewed in [78]). Severe pathophysiological markers for HCPS also include stimulation of IFNγ, TNF, VEGF and virus-specific CD8^+^ T cells (reviewed in [78]). The differential expression response in persistently infected rice rats is in striking opposition to the features predicted for susceptible hosts and RR29. First, the depletion of *Stat5b* and *Cnot1* are consistent with a push toward T_FH_ development. T_FH_ cells are a subset of CD4^+^ cells that have low IFNγ, IL-4 and IL-17 levels [79,80]. They localize to the B cell follicular zone and specifically support B cell expansion and differentiation. Importantly, T_FH_ cell differentiation is independent of Th1/Th2/Th17 differentiation (reviewed in [81]). A role for a T cell subset in immune protection against ANDV is consistent with the observation that total T cell depletion in the susceptible Syrian hamster model did not alleviate disease progression [82]. Secondly, depletion of *Cnot1* indirectly suggests that IFNγ levels may be reduced in rice rats rather than stimulated, as occurs with HCPS. Moreover, the enrichment of *Ppp1cc* and *Mff* support the hypothesis that the RIG-I anti-viral pathway is stimulated in persistently infected rice rats rather than inhibited, as has been shown for pathogenic hantavirus infections [83]. Lastly, depletion of *Gtfi2* appears to suggest stimulation of VEGFR production in persistently infected rice rats, indicating that hemostatic processes are modulated, although gross pathology was not evident.

## Conclusion

The transcriptomic profile of wild long-tailed pygmy rice rat spleens reveals a number of immunomodulatory genes that should contribute toward better understanding of the differences between reservoir animals and those that exhibit pathologic responses. The differential expression data are consistent with a scenario in which, upon sero-conversion to ANDV and resolution of acute infection, T_FH_ cells are stimulated, IFNγ levels are limited and Jun kinase-induced apoptotic markers are reduced in ANDV reservoir rice rats. Together, these markers could be demonstrative of limits of ANDV pathogenicity in this reservoir host. Further characterization of the acute and persistent infections will have to be done to fully understand the transition from acute to persistent infection.

## Supporting Information

S1 Fig Maximum likelihood trees of selected immune response genes. Defined protein domains were aligned using Muscle [84]. Maximum likelihood phylogenetic analysis with 1000 bootstrap iterations was performed in Geneious version 7.0.4. Bootstrap values are shown; bar indicates branch lengths. Bootstraps values < 50% are indicative of low confidence nodes. Missing proteins indicate that the given ortholog was unavailable. Aligned peptide lengths are in parentheses. A) Stat2 (759 aa) and Stat5b (264 aa). B) IL-1β (240 aa) and complement factor B (742 aa). C) Nos2 (148 aa) and Azgp1 (134 aa). (PDF)
(PDF)

S1 Table *De novo* transcript assembly. Transcripts representing unique annotated genes (n = 66,173) within the total assembly (n = 158078). (XLSX)
(XLSX)

S2 Table Immune response transcripts in Reactome database. Number and confidence level of each ortholog’s predicted immune modulatory pathway function. Output from Reactome v4.1.1 [29,30]. (XLSX)
(XLSX)

S3 Table DESeq data. Comparison group, gene name determined by BLAST orthology, Functional category, Log2FC, DESeq pvalue, FDR pvalue, BLAST details, RSEM counts per animal, DESeq output. Top panel, Persistent Infection (RR18/RR31); Bottom panel, RR29. (XLSX)
(XLSX)
