# Supplementary figures and images for: Transcriptome Markers of Viral Persistence in Naturally-Infected Andes Virus (Bunyaviridae) Seropositive Long-Tailed Pygmy Rice Rats
Source: PLoS One. 2015 Apr 9;10(4):e0122935. doi: 10.1371/journal.pone.0122935 (PMC4391749; doi:10.1371/journal.pone.0122935)

**A**

## Stat2

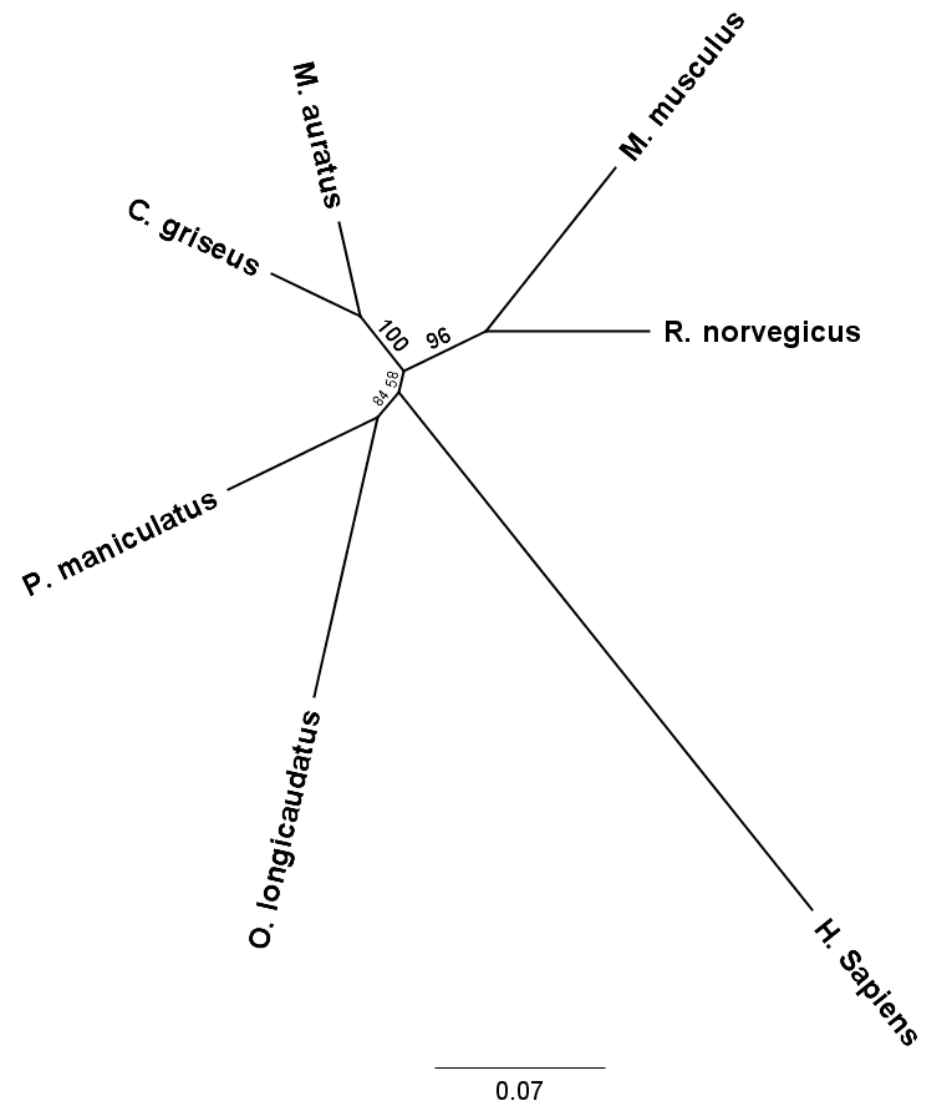

Stat5b

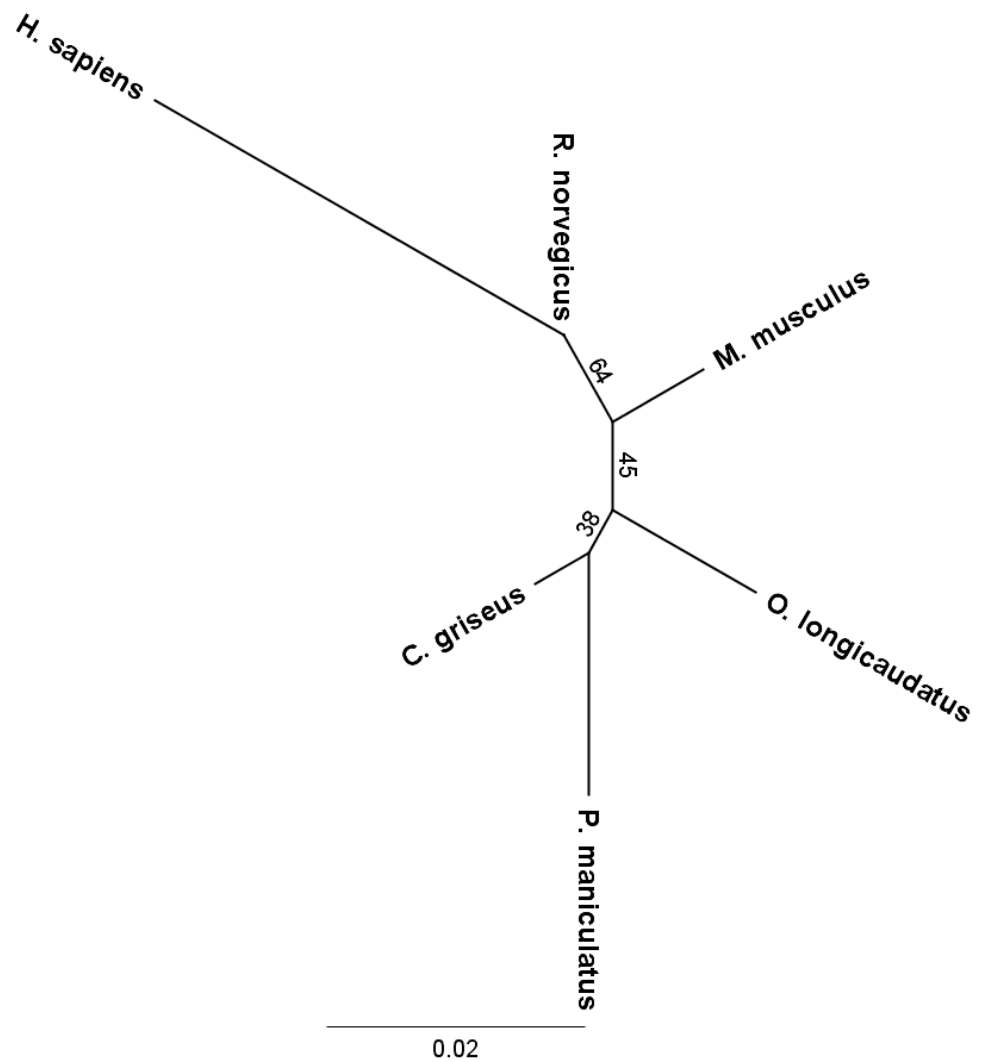

B

IL-1 beta

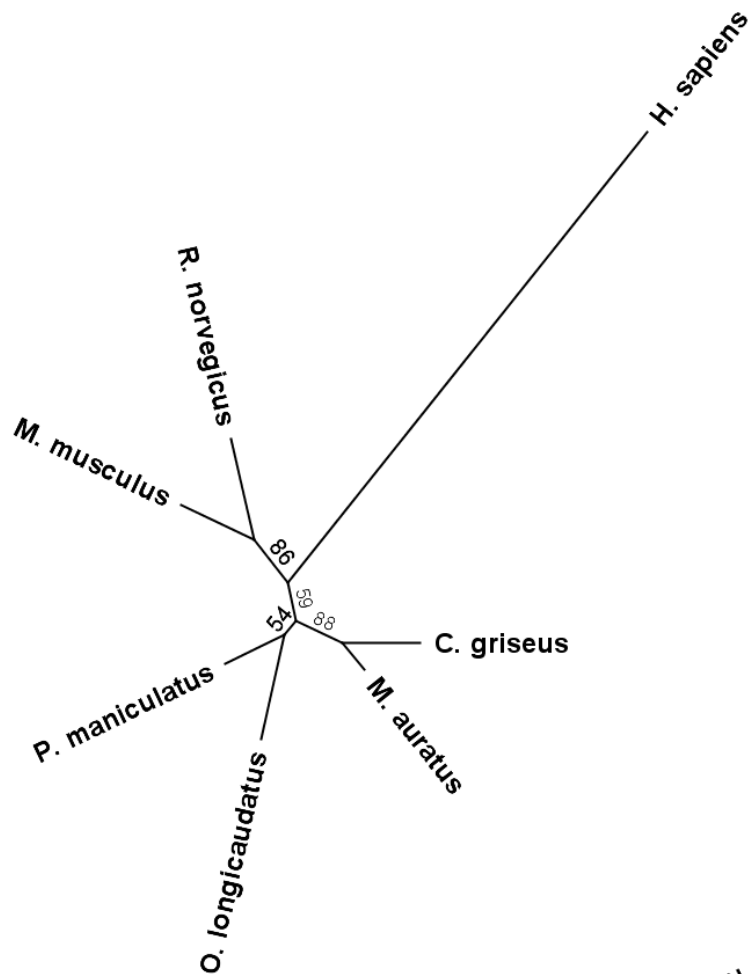

0.2

Complement factor B

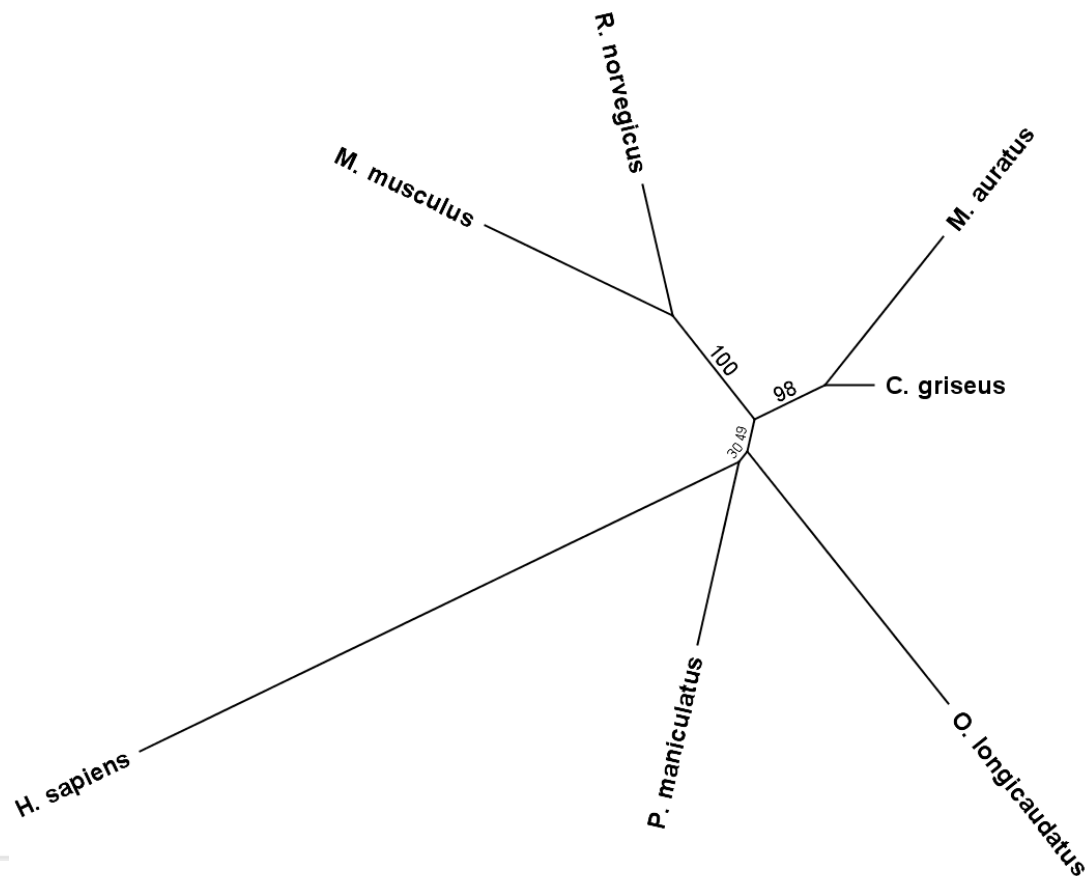

0.04

C

Nos2

Azgp1

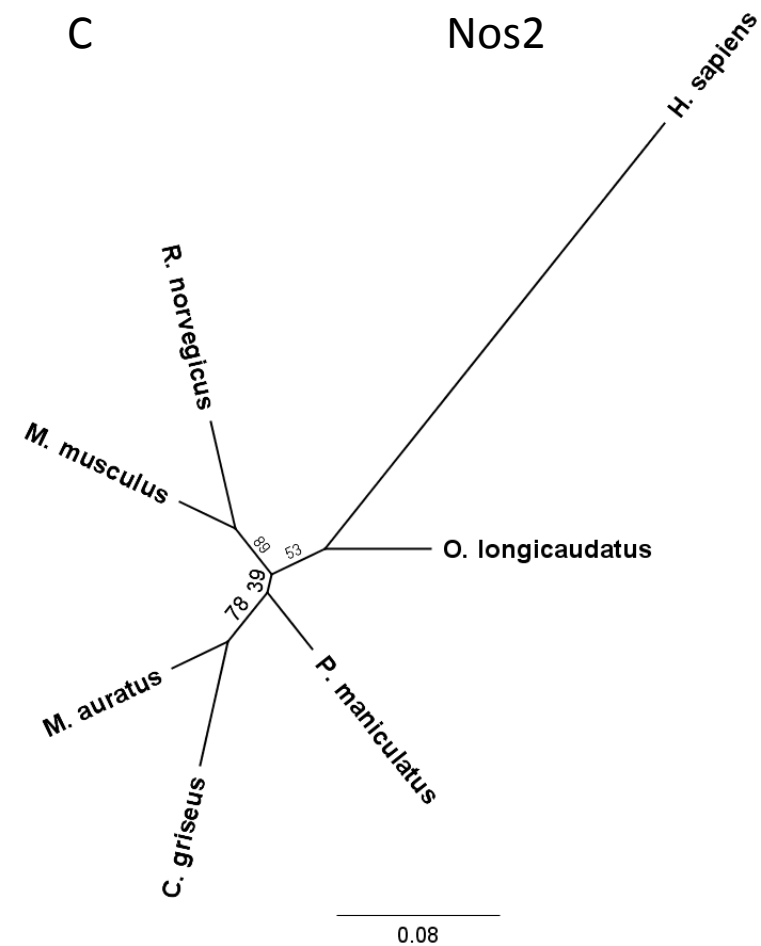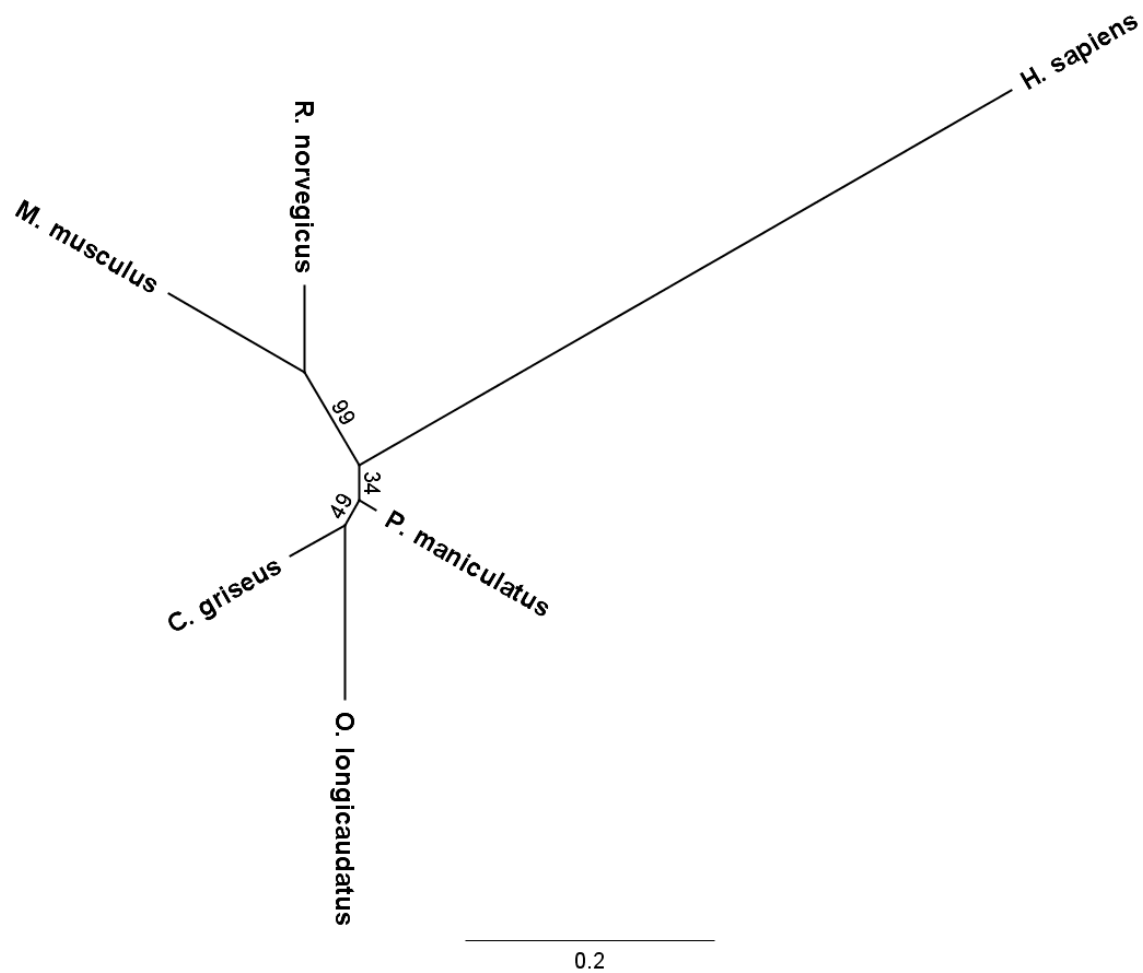

Supplement: S1 Fig — Defined protein domains were aligned using Muscle [84]. Maximum likelihood phylogenetic analysis with 1000 bootstrap iterations was performed in Geneious version 7.0.4. Bootstrap values are shown; bar indicates branch lengths. Bootstraps values < 50% are indicative of low confidence nodes. Missing proteins indicate that the given ortholog was unavailable. Aligned peptide lengths are in parentheses. A) Stat2 (759 aa) and Stat5b (264 aa). B) IL-1β (240 aa) and complement factor B (742 aa). C) Nos2 (148 aa) and Azgp1 (134 aa). (PDF) (PDF) [file pone.0122935.s001.pdf]
